# Associations of gender and serum total cholesterol with CD4+ T cell count and HIV RNA load in antiretroviral-naïve individuals in Addis Ababa

**DOI:** 10.1186/s12889-018-5852-4

**Published:** 2018-07-31

**Authors:** Melaku Adal, Rawleigh Howe, Desta Kassa, Abraham Aseffa, Beyene Petros

**Affiliations:** 1 Microbial, Cellular and Molecular Biology Department, Addis Ababa University, Addis Ababa, Ethiopia; 2 Armauer Hansen Research Institute, Addis Ababa, Ethiopia; 3 Ethiopian Public Health Institute, Addis Ababa, Ethiopia

**Keywords:** Gender, Total cholesterol, ART naïve, HIV RNA load, CD4+ T cell count

## Abstract

**Background:**

Males are more susceptible than females to infections due to the differences in endocrine-immune interactions. Furthermore, it is reported that lowering cell cholesterol impairs viral replication and infection in vitro. However, the production of oxysterols in vivo by oxidation of cholesterol may result in inhibition of HIV replication. Therefore, this study was designed to determine the associations of gender and serum total cholesterol with CD4+ T cell counts and/or WHO clinical stages, and HIV ribonucleic acid (RNA) load in antiretroviral therapy (ART) naive study population with known sero-positive time of stay in Addis Ababa.

**Methods:**

A cross-sectional study was conducted from February to August 2013 on 594 HIV-1 infected ART-naïve adult study participants in four hospitals Addis Ababa. CD4+ T-cell count, HIV RNA load, hemoglobin and fasting serum total cholesterol were determined. Socio-demographic characteristics, WHO clinical stages, and height and weight were collected from patients’ chart and triangulated by structured questionnaire. Pearson chi-square test, Spearman rank correlation and univariate and multivariate linear/logistic regression analyses were carried out to determine associations.

**Results:**

Mean HIV RNA load was found to be lower in women than in men (*p* < 0.05). CD4+ T cell count and serum total cholesterol were found to be significantly correlated with HIV RNA load (*p* < 0.01). Women were at lower risk of having higher HIV RNA load in comparison to men. In addition, having lower concentrations of serum total cholesterol was found to be independent predictor of higher HIV RNA load in comparison to those with higher concentrations of cholesterol in serum (*p* < 0.05). The multivariate binomial logistic regression also showed that the immune status was better in women than men, and in the presence of higher serum total cholesterol (*p* < 0.05).

**Conclusion:**

Gender and serum total cholesterol were found to be associated and independent predictors of HIV RNA load, and CD4+ cell count and/or WHO clinical stages. There is a significant lower HIV RNA load and better CD4+ T cell count in women and those study participants with higher serum total cholesterol.

**Electronic supplementary material:**

The online version of this article (10.1186/s12889-018-5852-4) contains supplementary material, which is available to authorized users.

## Background

According to the Joint United Nations Program on human immunodeficiency virus (HIV) and Acquired immune deficiency syndrome (AIDS) UNAIDS [1], an estimated 36.9 million people live with HIV globally, out of which about 25.8 million (70%) are in sub-Saharan Africa. HIV adult prevalence in Ethiopia was estimated at 1.5%, i.e.*,* 1.9% in females and 1.0% in males; the urban HIV prevalences was 5.2% in females and 2.9% in males; and rural prevalence was 0.8% in females and 0.5% in males. In addition, the prevalence of HIV in Addis Ababa was 6.0% in females and 4.3% in males [2]. The total estimated number of Ethiopians living with HIV was 793,700 [3], mainly infected by the subtype C [4, 5]. The World Health Organization (WHO) [6] currently recommends initiation of ART in people living with HIV/AIDS at any CD4+ T cell count regardless of the WHO clinical stages, giving priority to those with severe or advanced HIV disease (WHO clinical stages III/IV) or a CD4 T cell count ≤350 cells/mm^3^.

Total cholesterol could be used as important biomarker since lipids have a role in viral entry, uncoating, replication, protein synthesis, assembly, budding and infectivity [7, 8]. Replication of viruses is dependent upon regulation of the cellular cholesterol balance [9, 10] and endocrine-immunity interaction that differ along gender [11–13]. Viruses use membrane microdomains where viral cholesterol-rich region, receptors and/or coreceptors are localized called lipid rafts to infect the target cells [14, 15]. Nef of HIV inhibits activity of the ATP binding cassette transporter A1 (ABCA1) [16], and impairs cholesterol efflux [17, 18]. Nef also induces genes involved in cholesterol biosynthesis [17] and facilitate cholesterol delivery to lipid rafts [19]. Inhibiting its biosynthesis or depletion of cellular cholesterol by stimulation of cholesterol efflux through activation of ABCA1 suppresses HIV-1 infection and replication in vitro [20, 21].

During viral infection in vivo, the innate immune system produces interferons (IFNs) that are involved in up-regulation of interferon-stimulated genes (ISGs). Some of the ISGs are involved in production of oxysterols [22]. Cholesterol-25-hydroxylase (Ch25h) is one of the antiviral ISGs that can convert cholesterol to 25-hydroxycholesterol (25-HC). 25-HC inhibits viral entry by blocking membrane fusion between virus and cell [23, 24]. In addition, 25-HC controls sterol biosynthesis by feedback inhibition [25], and promotion of down-regulation of the enzymes involved in sterol biosynthesis [26].

Males are more susceptible than females to infections due to the differences in endocrine-immune interactions [11]. In support to this, studies showed that plasma HIV-1 RNA levels in women are lower than in men [27, 28]; and treatment with estrogen protects against the transmission of simian immunodeficiency virus (SIV) [12]. Beta-estradiol inhibited HIV-1 replication by inhibition infection through inducing higher expression of chemokines [29, 30].

The associations of gender and serum total cholesterol on HIV replication in vivo are not well investigated. Thus, this study was designed to investigate the association of gender and serum total cholesterol with CD4+ T cell count and viral load in ART naïve study participants. The outcome of the study is important to understand the association of gender and serum total cholesterol on HIV RNA load and CD4+ T cell count in vivo*.* This will have a potential importance to provide a basis for therapeutic strategies to control HIV-1 replication and infection.

## Methods

### Study setting, design and population

This cross-sectional study was conducted from February to August 2013 in Addis Ababa, Ethiopia, at All African Leprosy Rehabilitation and Training Centre (ALERT), St. Paul, Yekatit-12 and Zewditu Memorial Hospitals. A total of 594 study participants who are adults (age ≥18) and were ART naïve, enrolled for care in HIV care centres and waiting for ART drugs until they become eligible and willing to participate in the study were recruited consecutively. If the need for treatment of opportunistic infections arises, they were treated by chemoprophylaxis. This study was part of a bigger study that was planned to investigate the role of immune response on viral diversity of HIV-infected ART naïve patients. Institutional Research Ethics Review Committee (IRERC) of participating Institutions and the National Ethical Review Committee, Ministry of Science and Technology with reference number 3.10/004/2015 (Additional file 1: Figure S1) had approved the study. Enrolment of each study participant was done after giving full information and informed consent collected by anti-retroviral treatment nurses under close supervision of the principal investigator (MA). Patients with cognitive impairment and immediate intensive care requirement and pregnant women were excluded because we were bound to take only small amount of blood for those individuals that were not sufficient to the whole components of the study. Individuals who were taking drugs that could interfere with serum lipid levels during the study period were also excluded.

### Haematological and biochemical assays

Automated FACS counter (Becton and Dickinson, San Jose, CA, USA) was employed to determine CD4+ T cell count. Sysmex-21 (Sysmex, KX-21 N, Kobe, Japan) blood analyzer by noncyanide method was used to quantify hemoglobin. Cut values of < 12 g/dL in women and < 13 g/dL in men were considered anemic [31]. Enzymatic colorimetric method (Human diagnostics, HumanStar 180, Wiesbaden, Germany) was used to determine fasting serum cholesterol. Those study participants with serum cholesterol levels ≥200 mg/dL were defined as hypercholesterolemic [32].

### HIV RNA load determination

Abbott HIV-1 assay (Abbott Molecular Inc., Des Plaines, IL, USA) was used to determined HIV RNA load in 200 μL plasma.

### Questionnaire

Information on clinical, socio-demographic and anthropometry were collected from patients’ medical chart and triangulated by means of additional structured questionnaire on the day of blood sample collection (Additional file 2: Table S1).

### Anthropometric measurements

Body mass index (BMI) cut offs for nutritional status as thinness or acutely malnutrition (BMI < 18.5 kg/m^2^), normal (BMI = 18.5–24.9 kg/m^2^), overweight (BMI = 25.0–29.9 kg/m^2^) and obese (BMI ≥30 kg/m^2^) were used [33].

### Data analysis

The questionnaire and laboratory tests results (Additional file 3) were analyzed using STATA version 11.0 (Stata Corp, College station, Texas, USA) and GraphPad Prism version 5.03 (GraphPad software, California, USA). Frequency counts, percentages; mean ± standard deviation (SD) and median with inter quartile range (IQR) were presented. The independent t-test or analysis of variance (ANOVA) tests were used to compare means. Spearman rank order correlation between log viral load, CD4+ T cell count, BMI, cholesterol and hemoglobin level were done. In addition, Pearson chi-square to test the associations was used to analyze categorical data. Diagnostics performance of anemia, low serum total cholesterol and the two markers together for predicting CD4+ T cell count or WHO clinical stage, and HIV RNA load categories were analyzed using test agreement (kappa value) and Spearman rank order correlation. Risk factors for HIV RNA load were identified using univariate (for crude coefficient, β) and then multivariate linear regression analysis (for adjusted coefficient, β) after adjusting for potential cofounders. In addition, risk factors that were found to be statistically significant by chi-square test were analyzed using univariate for crude odds ratio (COR) and then for multivariate logistic regression analysis for adjusted odds ratio (AOR) to adjust for potential cofounders for CD4 T cell count and/or WHO HIV stage categories (CD4 < 200 and/or stages III/IV, CD4 < 350 and/or stages III/IV and CD4 < 500 and/or stages III/IV).

## Results

### Characteristics of the study population

As indicated in Table 1, 423 (71.2%) of the study participants enrolled in the study were women. The median age of the whole study participants was 34 years. In addition, the median ages 37 and 32 were for men and women, respectively (*p* < 0.001). The total proportion of study participants at AIDS stage or WHO clinical stages III/IV were 25.9% among which 14.4% were at WHO clinical stages III/IV. The median CD4+ T cell count ≥200 cells/mm^3^ for ~ 83% of the study participants were 357 cells/mm^3^ (IQR = 248–537); and had HIV RNA load for 500 study participants with detectable viral load of mean ± SD of 4.23 ± 0.83 log copies/mL. From the total study participants, 74 (12.3%) were found to have HIV RNA load below detectable limit (< 150 copies/mL) and it was not done for 10 (1.7%) due to sample limitation. Generally, the study participants were found 5.4% obese, 16.7% overweight and 15.1% undernourished. The prevalence of hypercholesterolemia in the study population was 16.7%. The anemia prevalence was 11.2% in the whole study population. It was 12.8% in men and 10.6% in women (*p* > 0.05). In addition, length of time when about 74% of study participants stay positive knowing their sero-positive status was more than a year.

**Table 1:** Characteristics of ART naïve study participants, February–September 2013, Addis Ababa, Ethiopia

Variables	Number	% (CI 95%)
Gender		
Male	171	28.8 (22.0–35.6)
Female	423	71.2 (66.9–75.5)
Marital status		
Never married	109	18.4 (11.1–25.7)
Married/living with partner	293	49.3 (43.6–55.0)
Divorced/widowed/separated	192	32.3 (25.7–38.9)
BMI (kg/m^2^)		
< 18.5	87	15.1 (7.6–22.6)
18.5–24.9	362	62.8 (57.8–67.8)
25.0–29.9	96	16.7 (9.2–24.2)
≥ 30	31	5.4 (−2.6–13.4)
Length of time stay positive (years)		
< 1	150	26.1 (19.1–33.1)
1–3	177	30.8 (23.8–37.4)
> 3	248	43.1 (37.1–49.5)
WHO clinical stage		
Stage 1	328	55.6 (50.2–61.0)
Stage 2	177	30.0 (23.2–36.8)
Stage ¾	85	14.4 (6.9–21.9)
CD4+ T cell count (cells/mm3)		
< 200	102	17.2 (9.9–24.5)
200–349	182	30.6 (23.8–37.3)
350–499	144	24.2 (17.2–31.2)
≥ 500	166	28.0 (21.2–34.8)
Hemoglobin level (g/dl)		
Non-anemic	507	88.8 (86.1–91.5)
Anemic	64	11.2 (3.5–18.9)
Cholesterol level (mg/dl)		
≤ 200	472	83.3 (79.9–86.7)
> 200	95	16.7 (9.2–24.2)
HIV RNA load (copies/mL)		
< 10,000	427	45.2(39.2–51.2)
≥ 10,000	157	54.8(49.3–60.3)
HIV RNA load in log copies/mL (mean ± standard deviation)	500	4.23 ± 0.83

### HIV RNA load correlated with some variables

**Fig. 1:**
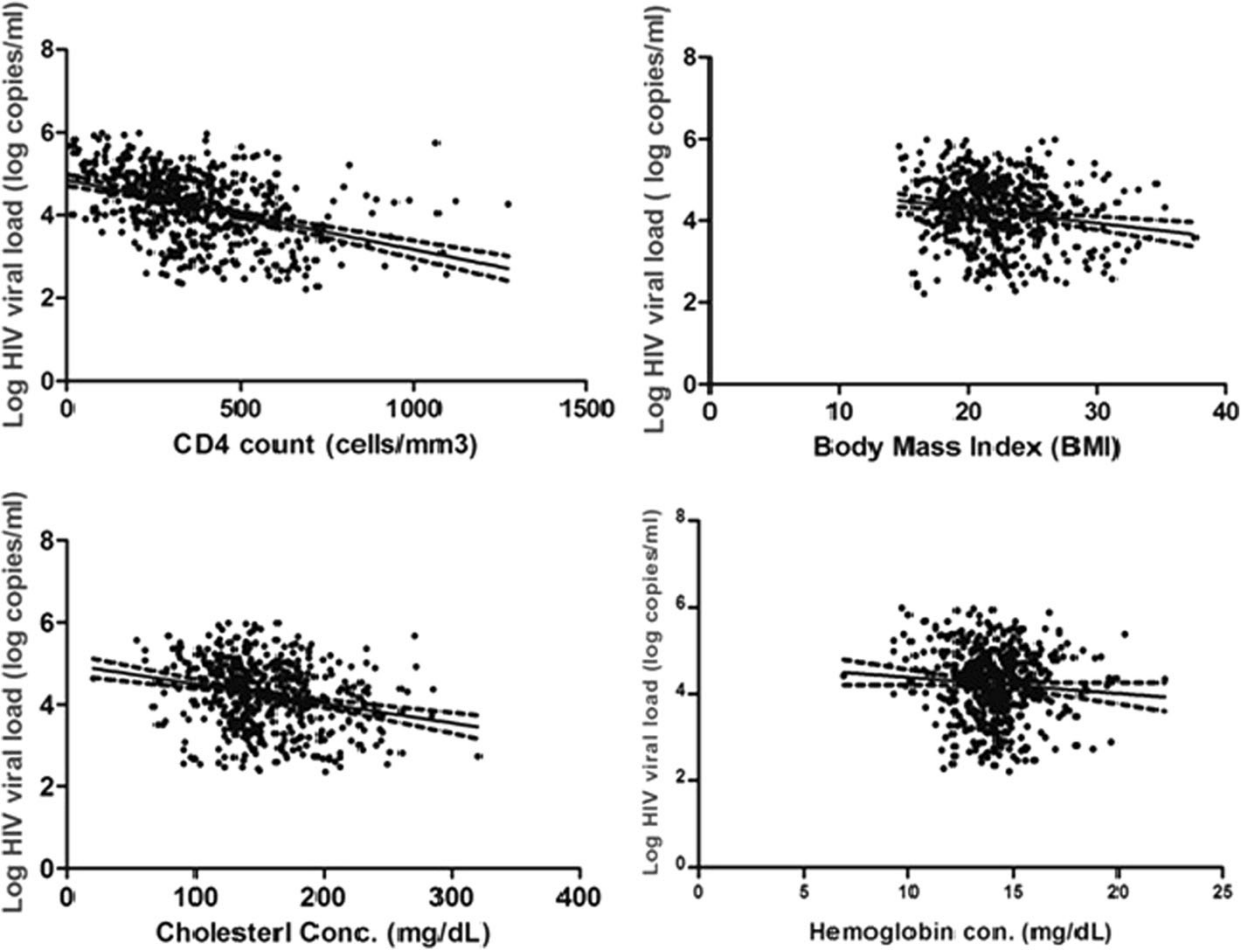
The correlation of log HIV RNA load with CD4+ T cell, BMI, total cholesterol and hemoglobin. The significant Pearson correlation (*p* < 0.01) between log HIV RNA load, and CD4+ T cell count, body mass index, serum total cholesterol (TC) and hemoglobin level of HIV-infected ART naïve study participants in Addis Ababa, Ethiopia

As indicated in Fig. 1 below, CD4+ T cell count (*r* = − 0.412, *p* < 0.01), body mass index (*r* = − 0.170, *p* < 0.01) and total cholesterol concentrations (*r* = − 0.249, *p* < 0.01) were found significantly correlated with HIV RNA load. However, hemoglobin concentrations were not significantly associated with HIV RNA load (*r* = − 0.084, *p* > 0.05).

### Independent predictors of HIV RNA load

Independent t-test revealed that mean HIV RNA load was found significantly higher in men, and in study participants with serum total cholesterol <200 mg/dL. In addition, the mean HIV RNA load was found to be different between CD4+ T cell count and/or WHO clinical stage categories, and anemic or normal (Table 2, *p* < 0.05).

**Table 2:** Associations of variables with HIV RNA load among ART-naïve study participants in Addis Ababa, Ethiopia

Variables	Log HIV RNA load (Mean ± SD)	Univariate, β (95% CI)	Multivariate, β (95% CI)
Gender			
Male	4.49 ± 0.76^a^	Ref.	Ref.
Female	4.11 ± 0.84	−0.38 (− 0.54, − 0.23)^a^	− 0.24 (− 0.40, − 0.07)^b^
CD4+ T cell count and/or stages III/IV			
≥ 500 and/or not stages III/IV	3.73 ± 0.82^a^	Ref.	Ref.
< 500 and/or stages III/IV	4.36 ± 0.79	0.63 (0.46, 0.80)^a^	0.56 (0.38, 0.73)^a^
Hemoglobin conc. (g/dL)			
Non-anemic	4.18 ± 0.81^c^	Ref.	Ref.
Anemic	4.68 ± 0.79	0.50 (0.27, 0.73)^a^	0.41 (0.18, 0.64)^b^
Total cholesterol conc. (mg/dL)			
≥ 200	3.85 ± 0.78	Ref.	Ref.
< 200	4.31 ± 0.82^c^	−0.46 (−0.67, − 0.25)^a^	− 0.25 (− 0.45, − 0.04)^c^
Variables	≥10,000, AOR (95% CI)	≥40,000, AOR (95% CI)	≥100,000, AOR (95% CI)
Gender			
Male	1.00	1.00	1.00
Female	0.51(0.33–0.79)	0.52(0.34–0.79)	0.64(0.41–1.08)^*^
CD4+ T cell count and/or stages III/IV			
≥ 500 and/or not stages III/IV	1.00	1.00	1.00
< 500 and/or stages III/IV	4.44(2.86–6.90)	5.29(2.90–9.64)	5.36(2.26–12.70)
Hemoglobin conc. (g/dL)			
Non-anemic	1.00	1.00	1.00
Anemic	2.85(1.44–5.66)	2.72(1.52–4.85)	2.46(1.34–4.51)
Total cholesterol conc. (mg/dL)			
≥ 200	1.00	1.00	1.00
< 200	2.22(1.32–3.74)	2.65(1.36–5.17)	2.35(0.97–5.69)^*^

*ANOVA* analysis of variance, *SD* standard deviation, and *CI* confidence interval; β = represents coefficients of univariate and multivariate linear regression; AOR = Adjusted odds ratio; ^a^, ^b^, ^c^ refers *p* value < 0.001, < 0.01 and < 0.05, respectively; and ^*^ = p not statistically significant

The association of each independent variable with dependent variable (log HIV RNA load) was analyzed by univariate linear regression analysis. As shown in Table 2, gender, CD4+ T cell count and/or WHO clinical stage categories, being anemic or normal, and being hypercholesterolemia or normal were found significantly associated with HIV RNA load (*p* < 0.05) and they were considered for multivariate linear regression analysis to determine the independent predictors of HIV RNA load.

The multivariate linear regression analysis showed that gender, CD4+ T cell count and/or WHO clinical stage categories, being anemic or normal, being hypercholesterolemia or normal were found to be independent predictors of HIV RNA load (Table 2, *p* < 0.05). Women are at lower risk of having higher HIV RNA load in comparison to men. Having higher total cholesterol was found to be associated with reduced HIV RNA load in comparison to those with lower total cholesterol in serum. In addition, study participants with CD4+ T cell count < 500 cells/mm^3^ and/or stages III/IV and who are anemic were found to be risk factors for increase of HIV RNA load. To prove whether gender, CD4 cell count < 500 cells/mm^3^ and/or WHO clinical stages III/IV, anemia and lower serum total cholesterol were also predictors of HIV RNA load along the three categories, binomial logistic regression analysis was done. CD4+ T cell count < 500 cells/mm^3^ and/or WHO clinical stages, and anemia were found to be significantly associated with the three HIV RNA categories. In addition, gender and low serum total cholesterol were found to be associated significantly with HIV RNA ≥10,000 and ≥ 40,000 copies/mL. However, gender and low serum total cholesterol were not found to be associated with HIV RNA ≥100,000 copies/mL (*p* > 0.05).

### Independent predictors of CD4+ T cell count and/or WHO clinical stages

**Fig. 2:**
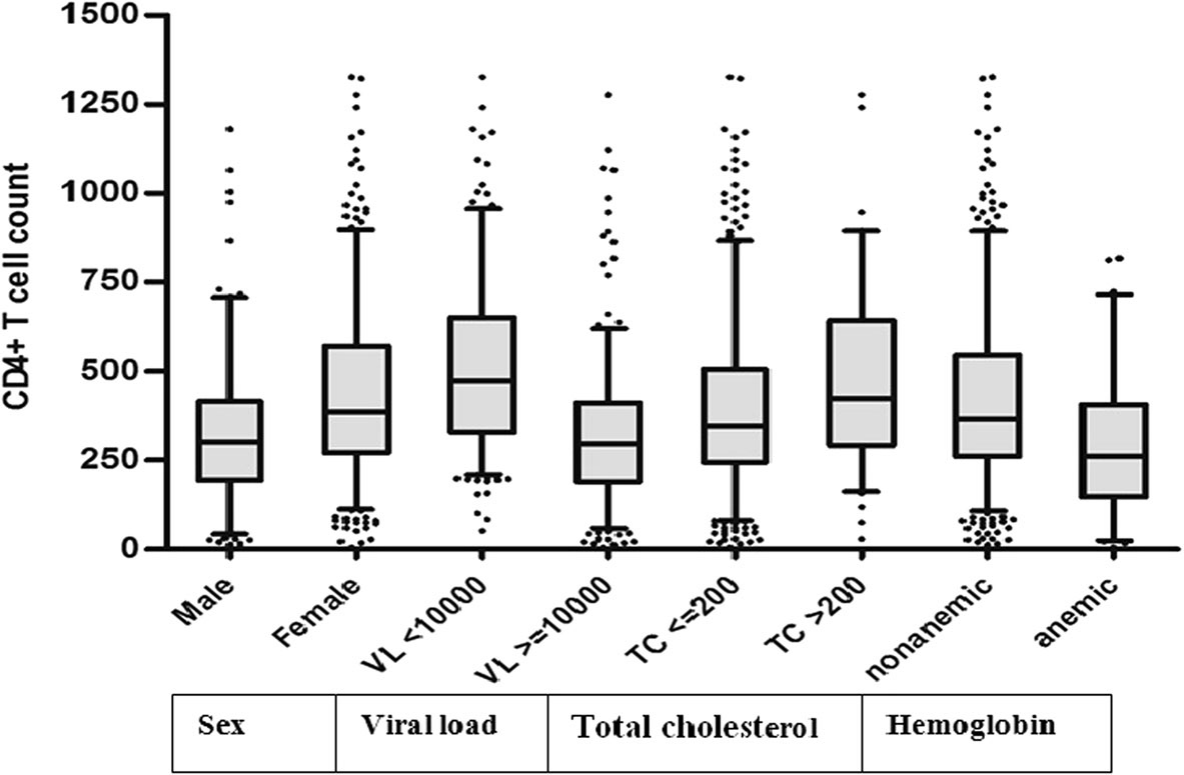
CD4+ T cell count by gender, HIV RNA load, serum total cholesterol (TC) and hemoglobin

As indicated in Fig. 2, Mann-whitney test between the median values CD4+ T cell count along gender, groups of HIV RNA load and serum total cholesterol, and being anemic or normal were done. The median (interquartile range) values of CD4+ T cell count were found to be [296(263–323) and 386(364–414), *p* < 0.001] for men and women, [385(364–409) and 195(164–249), *p* < 0.001] for HIV RNA load < 10,000- and ≥ 10,000 copies/mL, [352(326–365) and 457(368–502), *p* < 0.001] for serum total cholesterol < 200- and ≥ 200 mg/dL, and [258(206–313) and 366(354–389), *p* < 0.001] for being anemic and non-anemic, respectively.

As indicated in Tables 3 and 4, the Chi-square test or the univariate binomial logistic regression showed that gender, HIV RNA load, hemoglobin and serum total cholesterol levels were found to be associated with all the three categories of CD4+ T cell count and/or WHO clinical stages (*p* < 0.05). Multivariate binomial logistic regression analysis (Table 5) showed gender and HIV RNA load ≥10,000 to be independently associated with CD4+ T cell count and/or WHO clinical stage categories. In addition, anemia and low serum total cholesterol were found to be independently associated with CD4 T cell count < 200 and/or stages III/IV (*p* < 0.05). However, anemia and low serum total cholesterol were not significantly associated with CD4 T cell count < 350 and/or stages III/IV, and CD4 T cell count < 500 and/or stages III/IV (*p* > 0.05).

**Table 3:** Chi-square associations of variables with immune status among ART-naïve study participants in Addis Ababa, Ethiopia

Variables	Overall [Number (%)]	CD4 < 200 or stage III/IV [Number (%)]	CD4 < 350 or stage III/IV [Number (%)]	CD4 < 500 or stage III/IV [Number (%)]
Gender				
Male	171(28.8)	64(41.6) ^a^	111(35.5) ^a^	147(33.7) ^a^
Female	423(71.2)	90(58.4)	202(64.5)	289(66.3)
HIV RNA load (copies/mL)				
< 10,000	264(45.2)	27(17.9) ^a^	84(27.4) ^a^	149(34.8) ^a^
≥ 10,000	320(54.8)	124(82.1)	223(72.6)	279(65.2)
Hemoglobin conc. (g/dL)				
Non-anemic	507(88.8)	117(78.5) ^a^	252(83.7) ^a^	362(86.6) ^b^
Anemic	64(11.2)	32(21.5)	49(16.3)	56(13.4)
Total cholesterol conc. (mg/dL)				
> 200	95(16.6)	9(6.2)	34(11.4) ^a^	56(13.4) ^b^
≤ 200	476(83.4)	136(93.8) ^a^	264(88.6)	362(86.6)

Note: ^a^, ^b^ refer to *p* value < 0.001 and < 0.01, respectively

**Table 4:** Univariate associations of variables with immune status among ART-naïve study participants in Addis Ababa, Ethiopia

Variables	CD4 < 200 and/or stages III/IV, AOR (95% CI)	CD4 < 350 and/or stages III/IV, AOR (95% CI)	CD4 < 500 and/or stages III/IV, AOR (95% CI)
Gender			
Male	1.00	1.00	1.00
Female	0.45(0.31–0.67)	0.49(0.34–0.71)	0.35(0.22–0.57)
HIV RNA load (copies/mL)			
< 10,000	1.00	1.00	1.00
≥ 10,000	5.55(3.52–8.77)	5.00(3.51–7.12)	5.25(3.49–7.90)
Hemoglobin conc. (g/dL)			
Non-anemic	1.00	1.00	1.00
Anemic	3.33(1.96–5.67)	3.27(1.79–5.98)	2.80(1.3–6.03)
Total cholesterol conc. (mg/dL)			
> 200	1.00	1.00	1.00
≤ 200	3.82(1.87–7.81)	2.27(1.43–3.58)	2.21(1.40–3.50)

**Table 5:** Multivariate associations of variables with immune status among ART-naïve study participants in Addis Ababa, Ethiopia

Variables	CD4 < 200 and/or stages III/IV, AOR (95% CI)	CD4 < 350 and/or stages III/IV, AOR (95% CI)	CD4 < 500 and/or stages III/IV, AOR (95% CI)
Gender			
Male	1.00	1.00	1.00
Female	0.58(0.36–0.95)	0.60(0.36–0.99)	0.38(0.19–0.73)
HIV RNA load (copies/mL)			
< 10,000	1.00	1.00	1.00
≥ 10,000	3.89(2.34–6.46)	4.09(2.80–6.00)	4.49(2.69–7.49)
Hemoglobin conc. (g/dL)			
Non-anemic	1.00	1.00	1.00
Anemic	2.39(1.28–4.46)	1.73(0.86–3.56)^*^	1.67(0.65–4.28)^*^
Total cholesterol conc. (mg/dL)			
> 200	1.00	1.00	1.00
≤ 200	2.14(1.01–4.59)	1.53(0.90–2.54)^*^	1.38(0.75–2.56)^*^

Note: ^*^refers to *p* value not significant (*p* > 0.05)

Spearman’s rank order correlation (r) and *p*-value of each variable with CD4+ T cell count and/or WHO clinical stage, and HIV RNA load categories were done as shown in Additional file 4: Table S2. The correlations range from relatively small to moderate. Anemia, low serum total cholesterol and the combination of the two markers were found with significant but small correlation (*r* < 0.3, *p* < 0.05). Furthermore, CD4+ T cell count and/or WHO clinical stages, and HIV RNA load categories were also found to have significant moderate correlation between them (0.3 < *r* < 0.5, *p* < 0.01).

### Diagnostics performance of alternative markers to HIV disease progression

Diagnostics performance (sensitivity, specificity and predictive values) of HIV RNA load ≥10,000 or CD4 T cell count < 500 and/or Stage III/IV, anemia, lower serum total cholesterol, and combination of anemia and lower serum total cholesterol were done to determine disease progression as shown in Table 6. HIV RNA load ≥10,000 and CD4 T cell count < 500 and/or stages III/IV had a better sensitivity and specificity to determine disease progression. Low **s**erum total cholesterol alone, and the combination of anemia and lower serum total cholesterol showed high sensitivities of > 80.0 but low specificities. Lower serum total cholesterol was better than HIV RNA load ≥10,000 and CD4 T cell count < 500 and/or stages III/IV in its sensitivity, but low in its specificity to determine disease progression. Anemia did not have good diagnostic performance when used alone because the sensitivity was low even if the specificity was high. Low positive predictive values (PPV) and high negative predictive values (NPV) were observed in CD4 T cell count < 200 and/or stage III/IV. PPV value improved but NPV reduced in categories CD4 T cell count < 350 and/or stages III/IV, and CD4 T cell count < 500 and/or stages III/IV. However, it was the opposite of the CD4+ T cell count and/or WHO clinical stage categories that happened to the PPV and NPV in HIV RNA load categories (Table 6).

**Table 6:** Diagnostics performance of alternative biomarkers in reference with CD4+ T-cell count and/or WHO clinical stages, and HIV RNA load categories among ART-naïve study participants in Addis Ababa, Ethiopia

	Sensitivity	Specificity	PPV	NPV
Variables	CD4 < 200 and/or stages III/IV			
HIV RNA load ≥10,000	82.1	54.7	38.8	89.8
Anemia	21.5	92.4	50.0	76.9
Cholesterol < 200	93.8	20.2	28.6	90.5
Anemia and/or cholesterol < 200	85.1	24.5	28.3	82.4
	CD4 < 350 and/or stages III/IV			
HIV RNA load ≥10,000	72.6	65.3	70.1	68.1
Anemia	16.2	94.4	76.6	50.0
Cholesterol < 200	88.6	22.6	55.8	64.2
Anemia and/or cholesterol < 200	81.8	26.6	55.7	56.5
	CD4 < 500 and/or stages III/IV			
HIV RNA load ≥10,000	65.2	73.7	87.2	43.6
Anemia	13.4	94.8	87.5	28.6
Cholesterol < 200	86.6	25.5	76.1	41.1
Anemia and/or cholesterol < 200	80.5	29.1	75.8	35.1
	HIV RNA load ≥ 10,000			
CD4 < 500 and/or stages III/IV	87.2	43.6	65.2	73.7
Anemia	16.1	94.8	79.4	47.8
Cholesterol < 200	89.9	24.7	58.9	67.0
Anemia and/or cholesterol < 200	84.1	29.9	59.3	60.8
	HIV RNA load ≥ 40,000			
CD4 < 500 and/or stages III/IV	91.6	35.5	40.9	89.7
Anemia	20.2	93.1	58.7	70.7
Cholesterol < 200	93.5	22.4	36.8	87.6
Anemia and/or cholesterol < 200	86.9	26.7	36.6	80.8
	HIV RNA load ≥ 100,000			
CD4 < 500 and/or stages III/IV	92.0	30.6	21.5	94.9
Anemia	22.7	91.2	34.9	84.9
Cholesterol < 200	93.8	18.9	19.3	93.6
Anemia and/or cholesterol < 200	87.0	24.2	19.2	90.0

Note: *PPV* positive predictive value, *NPV* negative predictive value

## Discussion

This study was designed to investigate the association of gender and serum total cholesterol with CD4+ T cell count and/or WHO HIV stages III/IV, and the HIV RNA load in antiretroviral-naïve individuals with known time of staying HIV-positive in Addis Ababa, Ethiopia.

In concordance with other in vivo studies, this study showed that those study participants with high serum total cholesterol have lower HIV RNA load. This inhibition of HIV replication in study participants with high serum total cholesterol could be due to the production of oxysterols [24, 25]. In addition, the effect of ART is found impaired in hypocholesterolemic HIV-infected patients [34]. Viral infection induced IFNs up-regulate ISGs (for example, cholesterol-25-hydroxylase) and cause down-regulation of sterol biosynthesis to protect the cells [22, 26]. 25-HC as one of the oxysterols inhibits viral entry by blocking membrane fusion and controlling sterol biosynthesis [23, 24, 30]. However, in vitro studies showed discordance results in comparison with our study that higher serum total cholesterol is required for efficient viral replication. This role of cholesterol is clearly proved by inhibiting the cholesterol biosynthesis or depletion of cellular cholesterol content by stimulation of cholesterol efflux by ABCA1 that decrease virus entry and replication [15, 20, 21]. HIV plays increasing of cholesterol in cells through Nef protein that inhibits activity ABCA1 that impairs cholesterol efflux [16–18], and induces genes involved in cholesterol biosynthesis [17]. These differences in cholesterol role in vitro and in vivo indicate that it works differently in cell-lines in culture and human subjects.

This study also showed that HIV RNA load is higher in men than women. This may be because women produce higher antibody- and cell-mediated immune responses following either infection or vaccination than men that avoids infection and/or inhibits replication of the pathogens in the host [13]. With regard to the fluctuations of hormone level during menstrual cycle, in the follicular phase high estrogen levels and higher immunity may have protective effects against invading pathogens. During the luteal phase, however, high progesterone levels and reduced immunity may favor microbial invasion [14, 35]. The study done with estrogen treatment protects female rhesus macaques against the transmission of SIV by thickening of the genital tract mucosal tissue [12]. In addition, in vitro study demonstrated that beta-estradiol inhibited HIV-1 replication in human peripheral blood lymphocytes [29] by inhibiting target cell infection that involves cell-entry through higher expression of chemokines [30]. On the other hand, cells of the immune system in individuals with hypercholesterolemia had greater phagocytic activity, more circulating lymphocytes, more total T cells, more CD8+ T cells, more immunoglobulin production, more proliferation and differentiation, and migration of lymphocytes than from individuals with lower cholesterol levels [36–38]. This could be explained by the role of intermediates in the cholesterol-biosynthetic pathway and downstream oxysterol metabolites that have been found to influence diverse functions of cells of the immune system. However, significant gender differences in HIV RNA levels and CD4 counts are reported in HIV-infected children before the onset of puberty. These data indicate that intrinsic genetic differences between male and female individuals, unrelated to sex steroid hormone levels, influence HIV RNA level and CD4 parameters in HIV-infected individuals [39]. This may be possibly explained by epigenetic differences between the two genders [40, 41] even if the mechanism how epigenetic differences affect is not well clear.

Low serum total cholesterol and its combination with anemia showed high sensitivities of > 80.0 but low specificities in predicting disease progression (Table 6). The weak correlations, the low sensitivities and specificities, and the high fluctuation in PPVs and NPVs of this study may be partly explained by high background prevalences the general population of other non-HIV-related causes. For instance, anemia cannot distinguish between early and advanced HIV disease progression in high prevalence of infections and undernutrition [2, 42]. In addition, study participants were eligible for ART treatment when CD4+ T cell count < 200 cells/mm^3^ and/or WHO clinical stages III/IV according to the Ethiopian ART Guideline which was used during the study period [43]. In addition, about 83% of the study participants had CD4+ T cell count ≥200 cells/mm^3^ and only 14.4% were at WHO clinical stages III/IV during the study period. The effect of ART is impaired in hypocholesterolemic HIV-infected patients [34]. This may signify the potential use of low serum total cholesterol as predicting marker of ART efficacy in this era of test and treat. These biomarkers may enhance the performance of the physicians to examine the prognosis of the disease accurately among patients on ART. It has been also noted that switches in therapy will not be easy if physicians only had CD4+ T cell count because CD4+ T cell recovery is not high enough mainly in those who start ART late [6]. Using alternate biomarkers for monitoring ART efficacy is very useful tool through prolonging the interval of testing CD4+ T cell count and HIV RNA load. Basic laboratory testing and competent clinical monitoring of alternative biomarkers will thus be highly helpful under these circumstances [44, 45].

The study has some limitations. The recruitment of the study population did not include patients with cognitive impairment and immediate intensive care requirement. In addition, there may be false report of the some study participant that they are ART naïve. The other limitation of this study is the design. It was cross-sectional so that it is possible to identify associations but not causal relationships between risk and outcome variables. Therefore, there is need for prospective cohort or case control studies. In addition, serological evidence of hepatitis B/C, family history of dyslipidemia, changes in mood, depression, and factors related to lifestyle (smoking and physical inactivity) were unaccounted.

## Conclusions

Gender and serum total cholesterol were found associated with CD4+ T cell count and/or WHO clinical stage, and HIV RNA load categories. There is a significant lower HIV RNA load and better CD4+ T cell count in women and those study participants with higher serum total cholesterol. Therefore, further study should be done to verify the causal relationship between gender and serum total cholesterol with CD4+ T cell count and HIV RNA load. Therefore, documentation of such studies on the relationships of gender and serum total cholesterol with immunity and HIV replication may provide a basis for therapeutic strategies to control HIV replication.

## Additional files

Additional file 1: **Figure S1.** Ethical clearance. (PDF 582 kb)

Additional file 2: **Table S1.** Study participant demographic, socioeconomic, clinical and behavioral data collecting Questionnaire. (DOC 131 kb)

Additional file 3: Raw data. This additional file contains sociodemographic, clinical, immunological, virological, anthropometric and biochemical data of the study participants. (XLSX 66 kb)

Additional file 4: **Table S2.** Correlation of alternative biomarkers in reference with CD4+ T-cell count and/or WHO clinical stages, and HIV RNA load categories among ART-naïve study participants in Addis Ababa, Ethiopia. (DOCX 13 kb)

## Abbreviations

ABCA1: ATP binding cassette subfamily A member 1
AIDS: Acquired immune deficiency syndrome
ANOVA: Analysis of variance
AOR: Adjusted odds ratio
ART: Antiretroviral therapy
ATP: Adult Treatment Panel
BMI: Body mass index
CI: Confidence interval
COR: Crude odds ratio
EDHS: Ethiopian demographic and health survey
HC: Hydroxycholesterol
HIV: Human immunodeficiency virus
IQR: Inter quartile range
IRERC: Institutional Research Ethics Review committee
ISGs: Interferon-stimulated genes
NCEP: National Cholesterol Education Program
NPV: Negative predictive value
PPV: Positive predictive value
RNA: Ribonucleic acid
SIV: Simian immunodeficiency virus
TC: Total cholesterol
UNAIDS: Joint United Nations Program on HIV/AIDS
WHO: World Health Organization
