# Supplementary material for: Associations of gender and serum total cholesterol with CD4+ T cell count and HIV RNA load in antiretroviral-naïve individuals in Addis Ababa
Source: BMC Public Health. 2018 Jul 31;18:943. doi: 10.1186/s12889-018-5852-4 (PMC6069861; doi:10.1186/s12889-018-5852-4)
Supplement: Supplementary file 4 — Table S2. Correlation of alternative biomarkers in reference with CD4+ T-cell count and/or WHO clinical stages, and HIV RNA load categories among ART-naïve study participants in Addis Ababa, Ethiopia. (DOCX 13 kb) [file 12889_2018_5852_MOESM4_ESM.docx]

| **Table S3 .** Correlation of alternative biomarkers in reference with CD4+ T-cell count and/or WHO clinical stages, and HIV RNA load categories among ART-naïve study participants in Addis Ababa, Ethiopia. | | | | | | | | |
| --- | --- | --- | --- | --- | --- | --- | --- | --- |
| **Variables** | **CD4+ T cell count and/or WHO clinical stages III/IV** | | | | | | | |
|  | **CD4 <200 and/or stages III/IV** | | **CD4 <350 and/or stages III/IV** | | **CD4 <500 and/or stages III/IV** | | **Total CD4+ T cell count** | |
|  | **r** | **p** | **r** | **p** | **r** | **p** | **r** | **p** |
| HIV RNA load ≥10000 | 0.307 | <0.001 | 0.377 | <0.001 | 0.362 | <0.001 | -0.432 | <0.001 |
| Anemia | 0.199 | <0.001 | 0.173 | <0.001 | 0.121 | <0.01 | -0.187 | <0.001 |
| Cholesterol <200 | 0154 | <0.001 | 0.147 | <0.001 | 0.146 | <0.001 | -0.146 | <0.001 |
| Anemia and/or cholesterol <200 | 0.147 | <0.001 | 0.144 | <0.001 | 0.157 | <0.001 | -0.146 | <0.001 |
| **Variables** | **HIV RNA load** | | | | | | | |
|  | **≥10000** | | **≥40000** | | **≥100000** | | **Total HIV RNA load** | |
|  | **r** | **p** | **r** | **p** | **r** | **p** | **r** | **p** |
| CD4 <500 and/or stages III/IV | 0.362 | <0.001 | 0.311 | <0.001 | 0.319 | <0.001 | 0.333 | <0.001 |
| Anemia | 0.179 | <0.001 | 0.196 | <0.001 | 0.168 | <0.001 | 0.214 | <0.001 |
| Cholesterol <200 | 0.192 | <0.001 | 0.176 | <0.001 | 0.120 | <0.01 | 0.185 | <0.001 |
| Anemia and/or cholesterol <200 | 0.198 | <0.001 | 0.190 | <0.001 | 0.115 | <0.01 | 0.194 | <0.001 |
| **Note:** Classification of Correlation Co-efficient (r):- Up to 0.1: Trivial Correlation; 0.1-0.3: Small Correlation; 0.3-0.5: Moderate Correlation; 0.5-0.7: Large Correlation 0.7-0.9: very Large Correlation; 0.9- 1.0: Nearly Perfect correlation; 1: Perfect correlation. | | | | | | | | |
